# Pitavastatin nanoparticle-engineered endothelial progenitor cells repair injured vessels

**DOI:** 10.1038/s41598-017-18286-x

**Published:** 2017-12-22

**Authors:** Huanyun Liu, Pang Bao, Lufeng Li, Yuqing Wang, Chunxin Xu, Mengyang Deng, Jihang Zhang, Xiaohui Zhao

**Affiliations:** 1 Institution of Cardiovascular Research, Xinqiao Hospital, Third Military Medical University (Army Medical University), Chongqing, 400037 China; 2 Cardiovascular Department, First People’s Hospital of Chong Qing Liang Jiang New Zone, Chongqing, 401120 China; 3 Cardiovascular Department, The 180th Hospital of PLA, Quanzhou, Fujian, 362000 China

## Abstract

Endothelial progenitor cells (EPC) participate in vessel recovery and maintenance of normal endothelial function. Therefore, pitavastatin-nanoparticles (NPs)-engineered EPC may be effective in repairing injured vasculature. Pitavastatin-loaded poly(lactic-co-glycolic) acid (PLGA) NPs were obtained via ultrasonic emulsion solvent evaporation with PLGA as the carrier encapsulating pitavastatin. The effects and mechanism of pitavastatin-NPs on EPC proliferation *in vitro* were evaluated. Then, EPC that internalized pitavastatin-NPs were transplanted into rats after carotid artery injury. EPC homing, re-endothelialization, and neointima were evaluated by fluorescence labeling, evans Blue and hematoxylin/eosin (H&E) staining. Pitavastatin-NPs significantly improved EPC proliferation compared with control and pitavastatin group. Those effects were blocked by pretreatment with the pharmacological phosphoinositide 3-kinase (PI3K) blockers LY294002. After carotid artery injury, more transplanted EPC were detected in target zone in Pitavastatin-NPs group than pitavastatin and control group. Re-endothelialization was promoted and intimal hyperplasia was inhibited as well. Thus, pitavastatin-NPs promote EPC proliferation via PI3K signaling and accelerate recovery of injured carotid artery.

## Introduction

Vascular endothelial injury is the main pathophysiological basis for atherosclerotic diseases and restenosis after coronary intervention^1^. EPC are important for endothelial repair and therefore essential for a favorable outcome in these cases^2^. However, the quantity and biologic function of EPC are impaired in patients with multiple cardiovascular risks^3,4^. Thus, increasing EPC number by transplantation or improvement of EPC proliferation seems desirable strategy for vessel repair.

Statins are the most widely used agent for treatment of ischemic cardiovascular disease. They have cardioprotective effects independent of their lipid-lowering function that includes improving the biological function of EPC^5^. However, these pleiotropic effects require long-term administration of high statin dosages^6,7^, which is limited by relatively low oral bioavailability and therefore lead to an increased risk of adverse reactions^8,9^.

The emergence of nanotechnology has provided a new tool for improving *in vivo* drug delivery. Using biodegradable polymer materials as drug carriers, it is possible to alter the pharmacokinetic characteristics of encapsulated drugs or biomolecules to improve their efficacy and reduce adverse effects^10,11^. Drug-loaded nanoparticles can be internalized by cells via endocytosis and slowly release the drug over time; this technology can be applied to stem cell transplantation^12^.

Pitavastatin is a new-generation statin that has greater efficacy in terms of lipid regulation, improvement of endothelial function, and plaque regression. So, we investigate the effect and mechanism of pitavastatin-NPs on EPC proliferation and vessel repair.

## Results

### Characterization of pitavastatin-NPs

**Figure 1:**
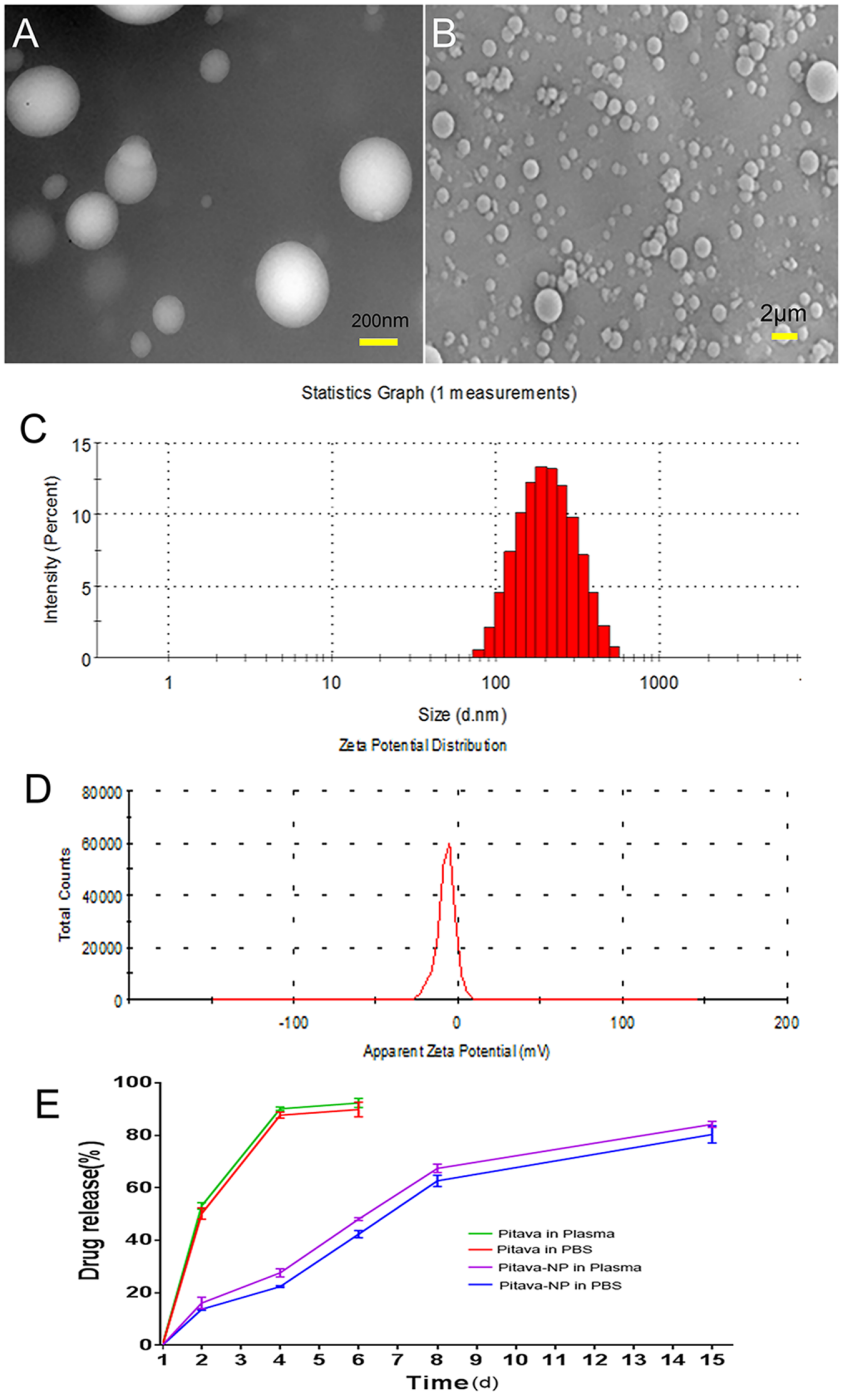
Characterization of pitavastatin-NPs. (**A**) Micrographs of pitavastatin-NPs obtained by TEM. (**B**) Micrographs of pitavastatin-NPs obtained by SEM. (**C**) Average particle size distribution profile of pitavastatin-NPs. (**D**) Zeta potential of pitavastatin-NPs. (**E**) *In vitro* cumulative drug release profile of pitavastatin-NPs in PBS and plasma (pH 7.4) incubated at 37 °C (n = 3).

The transmission electron microscopy(TEM) analysis revealed that NPs had a spherical shape and smooth surface. There was no obvious aggregation and no fusion (Fig. 1A). Similar observations were made by scanning electron microscopy (SEM) (Fig. 1B). Zetasizer Nano showed that the average particle size was (230.18 ± 44.97) nm(Fig. 1C). The polydispersity index was 0.1147 ± 0.047. Zeta potential was also analyzed by Zetasizer Nano and was anionic (−6.90 ± 1.22) mV (Fig. 1D). The concentration of pitavastatin in pitavastatin-NPs was determined by High-performance liquid chromatography (HPLC). The drug loading capacity was (10.00 ± 1.83)% and entrapment efficiency was (35.54 ± 5.40)%.

The *in vitro* drug release was tested in phosphate-buffered saline (PBS) and human plasma (37 °C, pH 7.4) (Fig. 1E). The results showed that pitavastatin release was more rapid in both PBS and human plasma. The amount of drug released in 3 days was (91.83 ± 4.82)% and (92.39 ± 3.28)%, respectively. In contrast, pitavastatin-NPs exhibited sustained and controlled release. The cumulative amount of drug released in 14 days reached (83.20 ± 5.63)% in PBS and (86.17 ± 2.83)% in plasma, respectively.

The stability of the PLGA nanoparticles is important for storage. In our study, there were no significant differences in the average particle size, Zeta potential, polydispersity index on 0, 10 and 20 days (P > 0.05) (Table 1).

**Table 1:** The average particle size, zeta potential and poly dispersity index of nanoparticles against storage time at 4 °C (P > 0.05) (n = 3, x̅  ± s).

Time(d)	average particle size(nm)	zeta potential(mV)	Polydispersity index
0	228.09 ± 35.35	−7.74 ± 0.91	0.2043 ± 0.053
10	233.29 ± 43.52	−7.63 ± 1.26	0.1734 ± 0.048
20	235.96 ± 48.71	−7.58 ± 0.48	0.1907 ± 0.018

### Cellular uptake and *in vitro* kinetics of PLGA nanoparticles in EPC

**Figure 2:**
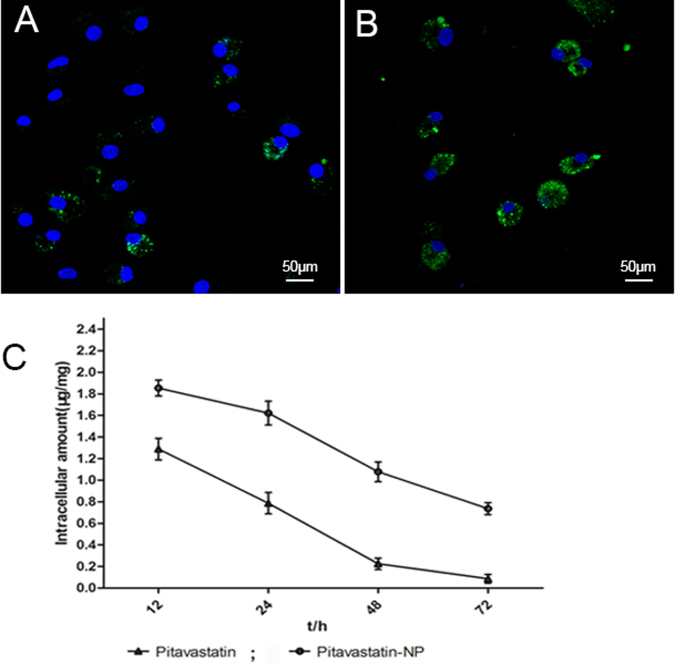
Cellular uptake of PLGA nanoparticle:Distribution of FITC-PLGA NPs in the cytoplasm of EPC after (**A**) 2 h and (**B**) 4 h of incubation;The cellular uptake kinetics of Pitavastatin in EPC (**C**).

EPC were incubated with the fluorescein isothiocyanate (FITC)-PLGA nano-emulsion (1 mg/ml) prepared using the emulsion solvent diffusion method for 2 or 4 h. Fluorescent granules were observed throughout the cytoplasm after 2 h of incubation, and their number increased at 4 h (Fig. 2A and B).

The accumulation of free and encapsulated pitavastatin in cells exhibited significant difference with time. Pitavastatin-NPs achieved a concentration of (1.854 ± 0.128) µg/mg of protein within 24 h, and continued to maintain higher during 72 hours. EPC treated with single pitavastatin for 24 hours showed a concentration of (1.288 ± 0.174) µg/mg of protein, which nearly can’t be detected after 72 hours. (Fig. 2C).

### PLGA has no effect on EPC viability

**Figure 3:**
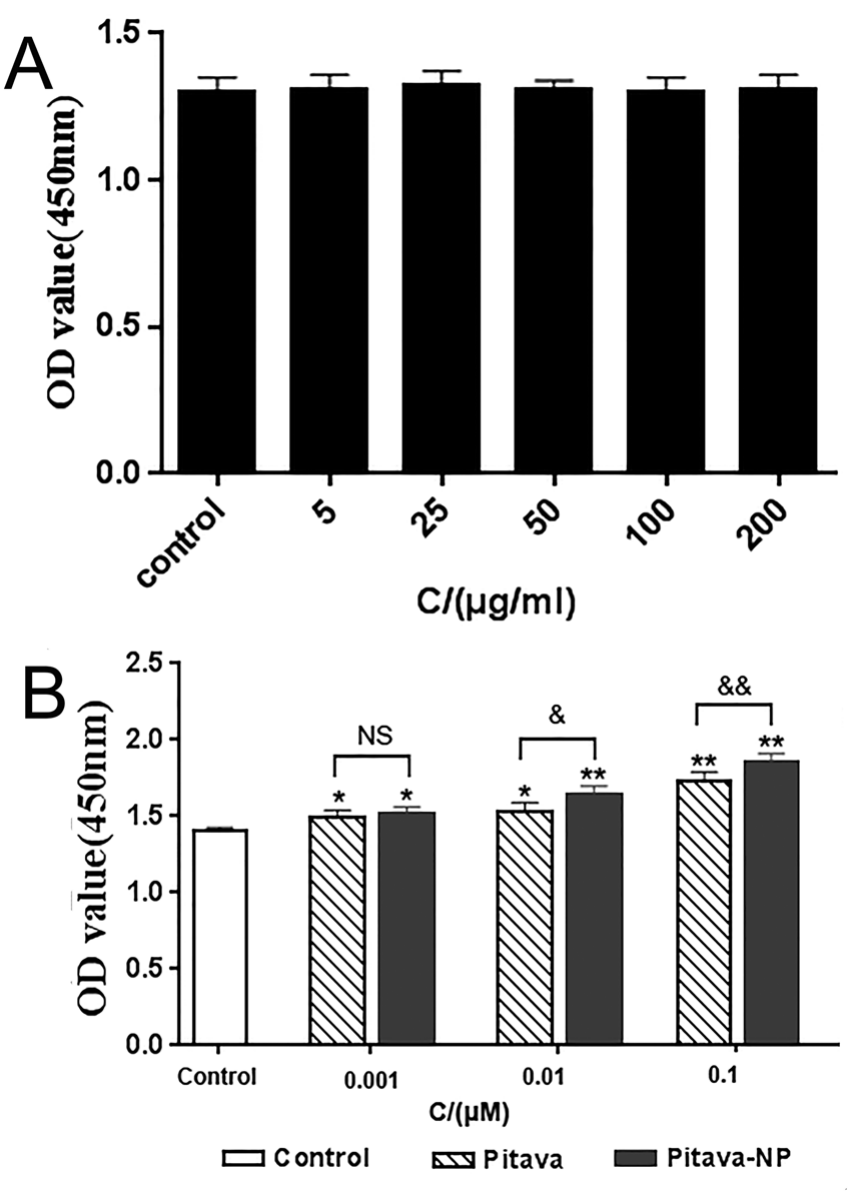
EPC viability and proliferation were analyzed by CCK8. (**A**) Effect of blank PLGA NPs on EPC viability (n = 9). (**B**) Effect of pitavastatin-NPs on EPC proliferation: *P < 0.05 vs. control, **P < 0.01 vs. control; ^&^P < 0.05 vs. 0.01 μM pitavastatin, ^&&^P < 0.01 vs. 0.1 μM pitavastatin; NS = not significant (n = 9).

The effect of blank PLGA NPs on EPC viability was evaluated with the Cell Counting Kit-8 (CCK8) assay. The absorbance of treated and control cells was similar among different concentrations groups(P = 0.495) (Fig. 3A).

### Pitavastatin-NPs promoted EPC proliferation

Pitavastatin-NPs promoted the EPC proliferation, as determined by CCK8 assay (0.001 µM pitavastatin-NPs vs. control group, 1.52 ± 0.04 vs. 1.41 ± 0.02, P < 0.05; 0.01 µM pitavastatin-NPs vs. control group, 1.64 ± 0.06 vs. 1.41 ± 0.02, P < 0.01; and 0.1 µM pitavastatin-NPs vs. control group, 1.86 ± 0.05 vs. 1.41 ± 0.02, P < 0.01). Compared to pitavastatin alone, pitavastatin-NPs increased proliferation at concentrations of 0.01 and 0.1 μM (0.01 μM pitavastatin-NPs vs. 0.01 μM pitavastatin, 1.64 ± 0.06 vs. 1.53 ± 0.06, P < 0.05; and 0.1 μM pitavastatin-NPs vs. 0.1 μM pitavastatin, 1.86 ± 0.05 vs. 1.73 ± 0.06, P < 0.01). There is no difference between 0.001 µM pitavastatin-NPs and pitavastatin group(0.001 μM pitavastatin-NPs vs. 0.001 μM pitavastatin, 1.52 ± 0.04 vs. 1.49 ± 0.05, P > 0.05) (Fig. 3B).

### PI3K signaling mediates pitavastatin-NP-induced EPC proliferation

**Figure 4:**
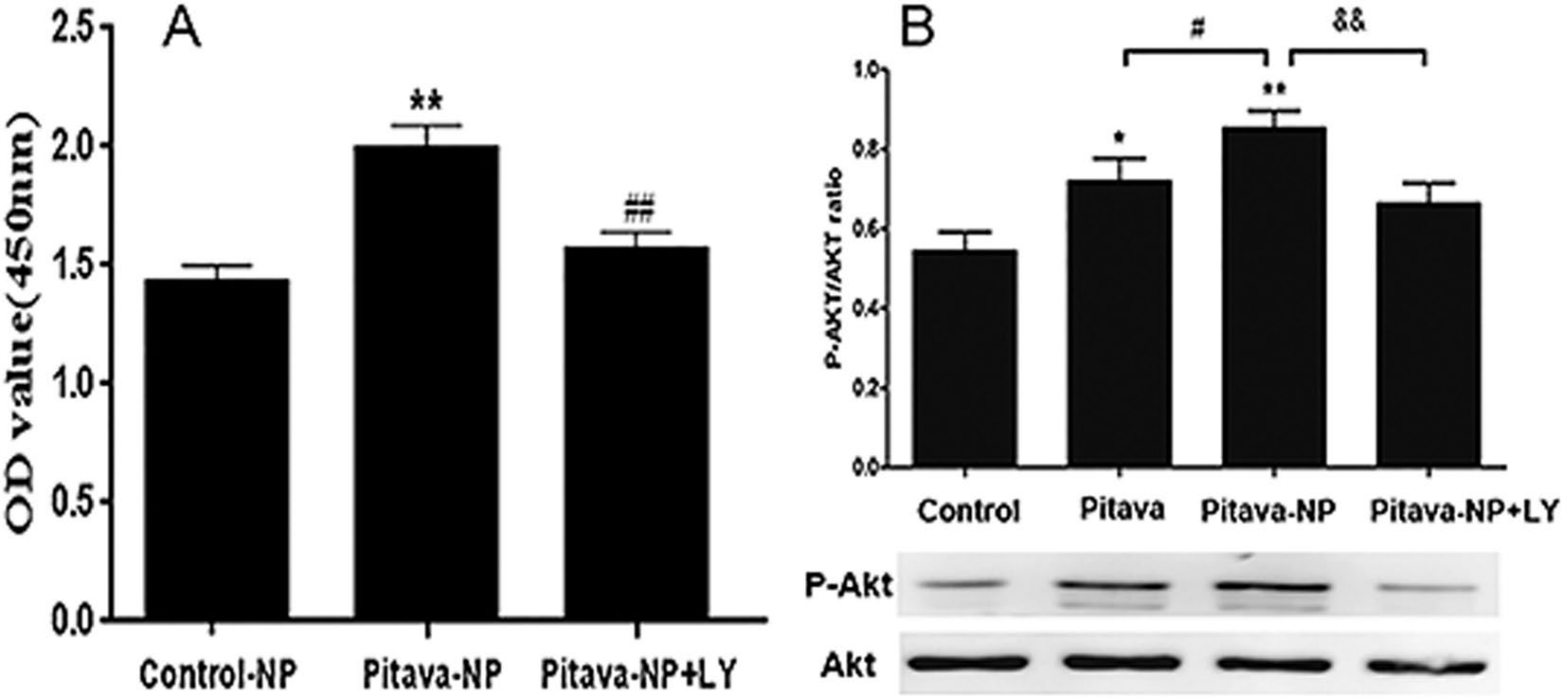
Role of PI3K/Akt signaling in pitavastatin-NP-induced EPC proliferation. (**A**) Effect of PI3K inhibitor treatment on pitavastatin-NP-induced EPC proliferation. **P < 0.01 vs. control-NP; ^##^P < 0.01 vs. pitavastatin-NP (n = 9). (**B**) Effect of PI3K inhibitor treatment on pitavastatin-NP-induced Akt phosphorylation in EPC. *P < 0.01 vs. control, **P < 0.01 vs. control; ^#^P < 0.05 vs. pitavastatin; ^&&^P < 0.01 vs. pitavastatin-NP (n = 4).

Western blot analysis revealed that pitavastatin-NPs enhanced Akt (Ser473) phosphorylation in EPC (0.1 μM pitavastatin-NPs vs. control group, 0.85 ± 0.13 vs. 0.55 ± 0.14, P < 0.01; and 0.1 μM pitavastatin-NPs vs. 0.1 μM pitavastatin, 0.85 ± 0.13 vs. 0.70 ± 0.17, P < 0.05) (Fig. 4B). However, treatment with PI3K inhibitor (LY294002) abrogated the pro-proliferative effect of pitavastatin-NPs [0.1 μM pitavastatin-NPs vs. 0.1 μM pitavastatin-NPs + LY294002 (10 μM), 1.99 ± 0.10 vs. 1.57 ± 0.09, P < 0.01] (Fig. 4A). This was associated with significantly decreased Akt (Ser473) phosphorylation (0.1 μM pitavastatin-NPs vs. 0.1 μM pitavastatin-NPs + LY294002 (10 μM), 0.85 ± 0.13 vs. 0.66 ± 0.15, P < 0.01) (Fig. 4B).

### Repair of injured vessels is induced by pitavastatin-NPs

#### Pitavastatin-NPs enhance EPC homing

**Figure 5:**
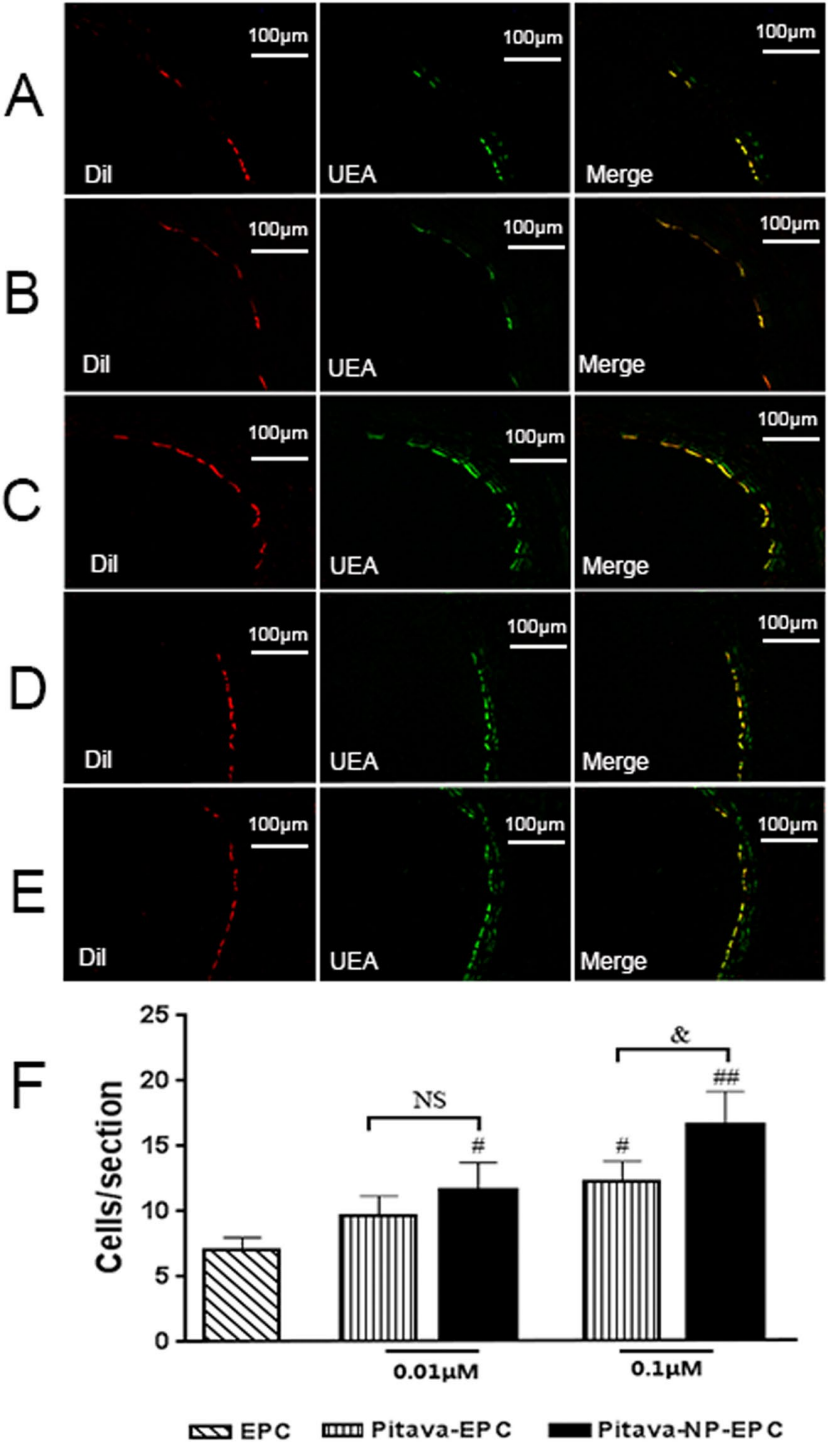
Effect of pitavastatin-NPs on the homing of EPC in damaged blood vessels. (**A**) EPC group, (**B**) 0.01 μM pitavastatin-EPC; (**C**) 0.01 μM pitavastatin-NP-EPC, (**D**) 0.1 μM pitavastatin-EPC, and (**E**) 0.1 μM pitavastatin-NP-EPC (n = 3). (**F**) Histogram of the number of EPC exhibiting homing in each group. ^#^P < 0.05 vs. EPC group, ^##^P < 0.01 vs. EPC group; ^&^P < 0.05 vs. 0.1 μM pitavastatin-EPC group; NS = not significant.

To determine whether EPC homed to damaged blood vessels and differentiated into endothelial cells, labeled EPC were transplanted to rats after carotid artery injury. DiI-labeled EPC, identified as red fluorescent cells, were seen lining the lumen via co-staining for the endothelial marker FITC-Ulex europaeus agglutinin I (UEA-I). Double-positive EPC were counted under an inverted fluorescence microscope(Fig. 5A–E). There was a significant increase of homing EPC in pitavastatin-NP group than control group (0.01 µM pitavastatin-NP-EPC vs. EPC group, 11.67 ± 2.08 vs. 7.00 ± 1.00, P < 0.05; and 0.1 µM pitavastatin-NP-EPC vs. EPC group, 16.67 ± 2.52 vs. 7.00 ± 1.00) (P < 0.01). In addition, the number of homing EPC was higher in the pitavastatin-NP than in the pitavastatin group (0.1 µM pitavastatin-NP-EPC vs. 0.1 μM Pitavastatin-EPC group, 16.67 ± 2.52 vs. 12.33 ± 1.53, P < 0.05) (Fig. 5F).

#### Pitavastatin-NPs promote re-endothelialization of injured vessels

**Figure 6:**
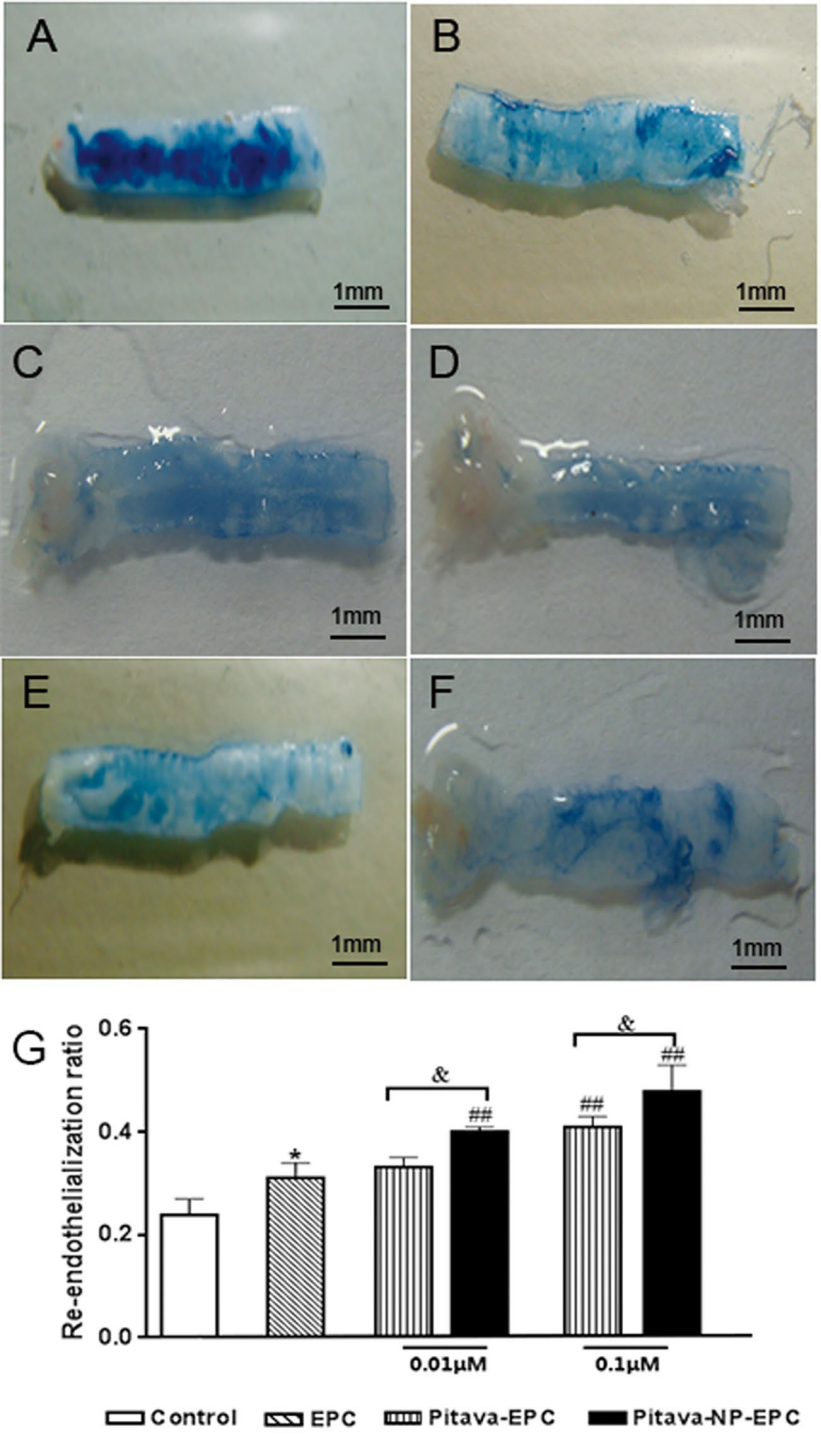
Effect of pitavastatin-NPs on re-endothelialization of damaged blood vessels. (**A**) Control, (**B**) EPC, (**C**) 0.01 μM pitavastatin-EPC, (**D**) 0.01 μM pitavastatin-NP-EPC, (**E**) 0.1 μM pitavastatin-EPC, and (**F**) 0.1 μM pitavastatin-NP-EPC (n = 3). (**G**) Histogram of re-endothelialization rate. *P < 0.05 vs. control; ^##^P < 0.01 vs. EPC group; ^&^P < 0.05 vs. pitavastatin-EPC group; NS = not significant.

Sections of *injured vessels* were stained with Evans Blue and re-endothelialization rate was calculated. Transplantation of EPC promoted re-endothelialization (EPC vs. surgical injury alone group, 0.31 ± 0.03 vs. 0.24 ± 0.03, P < 0.05). The rate of re-endothelialization after pitavastatin or pitavastatin-NP pre-treatment were higher than single EPC transplantation group (0.01 µM pitavastatin-NP-EPC vs. EPC group, 0.40 ± 0.01 vs. 0.31 ± 0.03, P < 0.01; 0.1 µM pitavastatin-EPC vs. EPC group, 0.41 ± 0.02 vs. 0.31 ± 0.03, P < 0.01; and 0.1 µM pitavastatin-NP-EPC vs. EPC group, 0.48 ± 0.05 vs. 0.31 ± 0.03, P < 0.01). Pitavastatin-NPs promoted re-endothelialization when compared to pitavastatin alone (0.01 µM pitavastatin-NP-EPC vs. 0.01 µM pitavastatin-EPC, 0.40 ± 0.01 vs. 0.33 ± 0.02, P < 0.05; and 0.1 µM pitavastatin-NP-EPC vs. 0.1 µM pitavastatin-EPC, 0.48 ± 0.05 vs. 0.41 ± 0.02, P < 0.05) (Fig. 6).

#### Pitavastatin-NPs inhibit intimal hyperplasia

**Figure 7:**
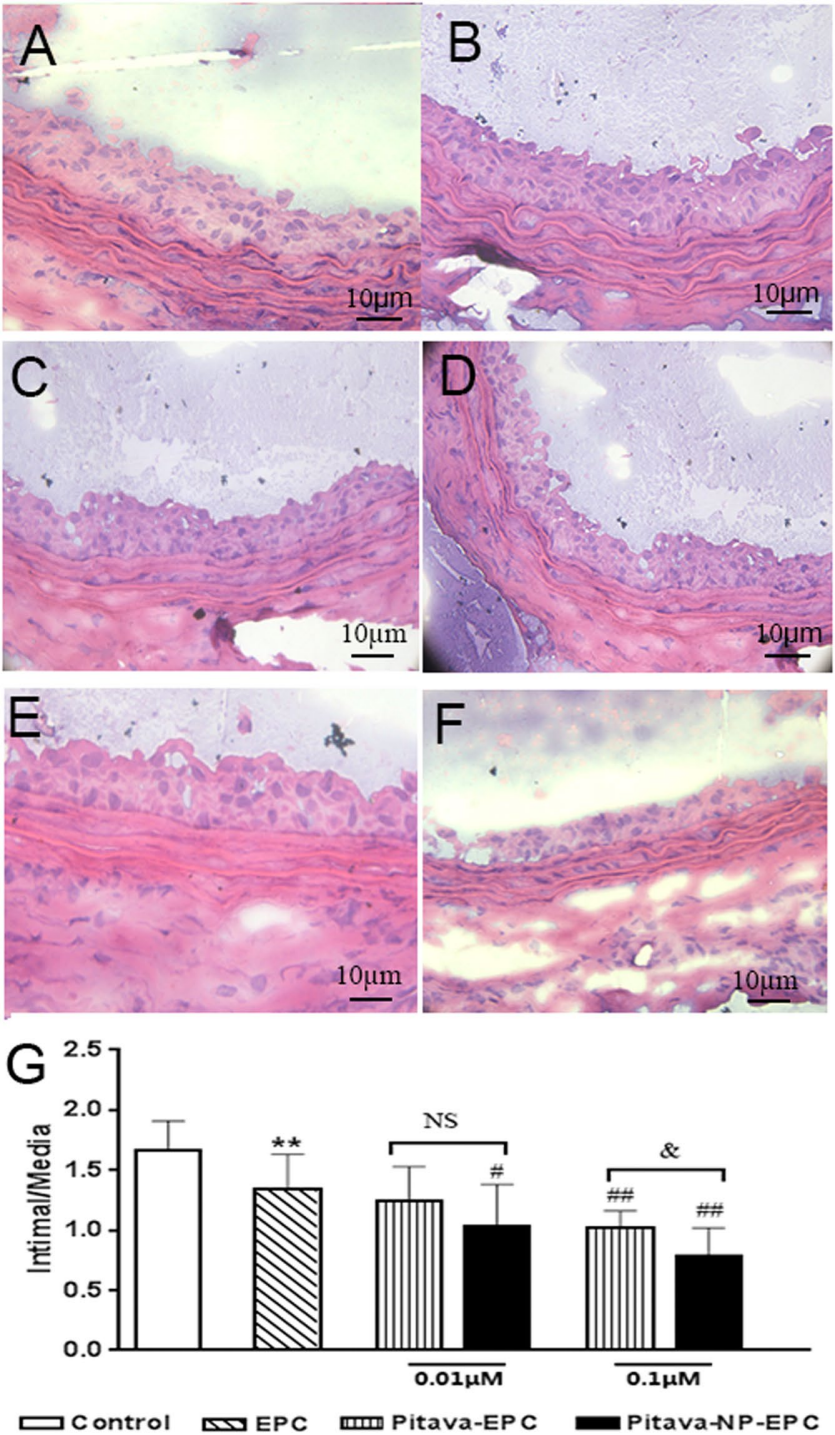
Effect of pitavastatin-NPs on intimal hyperplasia in injured blood vessels. (**A**) Control, (**B**) EPC, (**C**) 0.01 μM pitavastatin-EPC, (**D**) 0.01 μM pitavastatin-NP-EPC, (**E**) 0.1 μM pitavastatin-EPC, and (**F**) 0.1 μM pitavastatin-NP-EPC (n = 6). (**G**) I/M ratio. **P < 0.01 vs. control; ^#^P < 0.05 vs. EPC group; ^##^P < 0.01 vs. EPC group; ^&^P < 0.05 vs. pitavastatin-EPC group; NS = not significant.

Arterial intimal hyperplasia was observed by H&E staining and evaluated using the I/M ratio. A lower ratio was observed in EPC group than surgical injury group (EPC vs. surgical injury only group, 1.34 ± 0.29 vs. 1.67 ± 0.24, P < 0.01), while the I/M value of the pitavastatin group was lower than that of the EPC group (0.01 µM pitavastatin-NP-EPC vs. EPC group, 1.03 ± 0.35 vs. 1.34 ± 0.29, P < 0.05; 0.1 µM pitavastatin-EPC vs. EPC group, 1.02 ± 0.14 vs. 1.34 ± 0.29, P < 0.01; and 0.1 µM pitavastatin-NP-EPC vs. EPC group, 0.79 ± 0.23 vs. 1.34 ± 0.29, P < 0.01). Moreover, the ratio in the 0.1 µM pitavastatin-NP-EPC group was lower than the same concentration of pitavastatin group (0.1 µM pitavastatin-NP-EPC vs. 0.1 µM pitavastatin-EPC group, 0.79 ± 0.23 vs. 1.02 ± 0.14, P < 0.05) (Fig. 7).

## Discussion

This study for the first time reported that pitavastatin promoted EPC proliferation via PI3K signaling and pitavastatin nanoparticle-engineered EPC accelerated vessel recovery after carotid artery injury in rats.

Strategies that enhance the number of EPC may facilitate angiogenesis and recovery of injured endothelium. Pitavastatin is found to promotes the EPC proliferation^13^. However, the mechanism is not very clear.

There are multiple mechanisms involved in statin-induced EPC proliferation modulation. In previous study, pitavastatin had been shown to induce EPC proliferation via increased the endothelial nitric oxide synthase (eNOS) and vascular endothelial growth factor (VEGF) expression in high-risk patients including those with hyperlipidemia and T2DM^13^. Steinmetz *et al*. reported that atorvastatin promote EPC proliferation by activating the receptor activator of NF-kappaB ligand (RANKL)^14^. Cerda *et al*. found both atorvastatin and simvastatin down-regulate the expression of miR-221^15^. Furthermore, miR-221 has been observed to impair proliferation of CD34-positive haematopoietic progenitor cells by reducing expression of c-kit receptor factor^16^. However, the PI3-kinase enzymes are widely expressed and also play crucial roles in cell proliferation. We and others have reported that PI3K signaling is important for the regulation of EPC proliferation, suggesting a potential mechanism^17,18^. First, PI3K catalyse the conversion of phosphatidylinositol-3, 4-bispho- sphate (PIP2) to phosphatidylinositol-3, 4,5-trisphosphate (PIP3)^19^. Then, PIP3 recruits protein kinase B, also known as Akt to the plasma membrane and serves as a multifunctional regulator of cell biology^20^. Llevadot *et al*. first reported simvastatin can rapidly activate Akt in EPC, enhancing their proliferative and migratory activities^21^. In this study, we found that pitavastatin improved EPC proliferation and enhanced Akt phosphorylation. However, treatment with the PI3K blocker-LY294002 inhibited pitavastatin-induced EPC proliferation, suggesting that pitavastatin regulate EPC proliferation via PI3K signaling.

Previous studies have shown that pitavastatin-NPs can be endocytosed by endothelial cells and have long-lasting effects^22,23^. In our study, pitavastatin-NPs were obtained via ultrasonic emulsion solvent evaporation. The average particle size of the pitavastatin-NPs was similar to previous reported size range, which have been shown to effectively penetrate cells and tissue vasculature^24^. Also, pitavastatin-NPs uptaken by EPC continuously released the drug along with PLGA degradation and therefore significantly improved EPC proliferation than pitavastatin alone.

Nanotechnology has already been used for the local administration of drugs for vessels repair. A previous study showed that a single intramuscular injection of pitavastatin-NPs resulted in drug targeting to ischemic skeletal muscle tissue—mainly endothelial cells—in a mouse model. Pitavastatin was continuously released with PLGA hydrolysis, thereby promoting vascular regeneration and reperfusion of ischemic tissues; notably, the effect was greater than that of a single dose of pitavastatin. Similar findings were reported in a rabbit model of chronic lower limb ischemia, which showed that pitavastatin-NPs were 100–300 times more effective than pitavastatin alone in terms of enhancing revascularization of ischemic tissues and persisted in living cells for up to 2–4 weeks^22,23^.

We found that after internalizing pitavastatin-NPs, more EPC accumulated in target zone of injured vessel, effectively promoted the endothelialization and prevented intimal hyperplasia. The underlying mechanism may involve slow hydrolysis of pitavastatin-NPs. Interestingly, it was reported that cells can release a fraction of drug-loaded NPs via exocytosis, which can then act on adjacent cells^25^. Therefore, in the microenvironment of injured blood vessels, transplanted EPC may act on smooth muscle cells via a paracrine mechanism releasing pitavastatin-NPs.

In summary, we found that pitavastatin promote EPC proliferation via PI3K signaling. Furthermore, pitavastatin nanoparticle-engineered EPC accelerate vessel recovery, indicating a promising treatment for vascular injury disease.

## Materials and methods

### Preparation of nanoparticles

NPs were prepared using a modified solvent-evaporation method^22^. Briefly, 150 mg of PLGA (75:25, Dai gang biotechnology, China) and 50 mg of pitavastatin (Abcam, U.S.A.) or 5 mg fluorescein isothiocyanate (FITC) (Sigma, U.S.A.) were dissolved in 2 ml dichloromethane (Kelong, China), and the resultant solution was slowly mixed with 1 ml of anhydrous ethanol (Kelong, China). The mixed solution was then added to 30 ml of 2% poly (vinyl alcohol) solution (Kelong, China), and emulsification was carried out for 8 min (amplitude: 50%, pulse ratio: 4:2) using a probe-type sonicator (YC-750, Arstevip, U.S.A.). The sample was stirred overnight with a magnetic stirrer (400 rpm) (MS-H280-PRO, China). Solidified NPs were centrifuged at high speed (4 °C, 22,000 rpm, 30 min), and the sample was washed twice with double-distilled water, freeze-dried for 24 h, and stored in a desiccator until use.

### Characterization of pitavastatin-NPs

A small amount of pitavastatin-NPs were weighed and dissolved in distilled water to obtain nanosuspensions (0.5 mg/ml). After ultrasonic dispersion, the average particle size, polydispersity index and zeta potential (surface charge) of pitavastatin-NPs were analyzed by Zetasizer Nano (Zetasizer NanoZS90, Malvern, U.K.). Drops of the nanosuspension were placed on the adhesive side of aluminum foil, dried, and sputter-coated in gold under vacuum, and particle morphology was examined by SEM (S-3400N, Hitachi, Japan). In addition, drops of the nanosuspensions were placed on a copper mesh and after drying, NP morphology was visualized by TEM (HT7700, Hitachi, Japan).

HPLC (waters e2695, water, U.S.A.) was used to assess drug-loading capacity, entrapment efficiency, and *in vitro* drug release. The HPLC conditions were as follows: bridge C18 column (150 mm × 4.6 mm, 3.5 μm); mobile phase: acetonitrile-0.01 mol/l and potassium dihydrogen phosphate (50:50); flow rate of mobile phase: 0.6 ml/min; injection volume: 10 μl; column temperature: 25 °C; ultraviolet detection wavelength: excitation wavelength = 245 nm, emission wavelength = 420 nm. The supernatant and rinsing solution obtained during preparation were used as test samples. Pitavastatin content was determined after diluting the sample by a known factor. Entrapment efficiency and drug-loading capacity were then calculated as follows: entrapment efficiency = 1 − W_1_/W_0_ × 100%; drug loading capacity = 1 − W_1_/W_2_ × 100%, where W_0_ (mg) is the amount of pitavastatin added during preparation; W_1_ (mg) is the pitavastatin content in the supernatant and rinsing solution; and W_2_ (mg) is the mass of NPs after freeze drying.

*In vitro* release analysis was performed in phosphate-buffered saline (PBS, Sigma, U.S.A.) and human plasma (37 °C, pH 7.4). Briefly, pitavastatin-NPs (25 mg) or pitavastatin (0.25 mg) was dispersed in 10 ml media. The sample was placed in a sealed dialysis bag in a 100-ml stoppered glass container containing PBS or plasma. Vibration dialysis was performed in a thermostatic shaker (37 °C, 100 rpm), and the dialysate (10 ml) was collected on days 1, 3, 5, 7, and 14. Fresh media was added to maintain the total volume at 100 ml. The amount of pitavastatin in the dialysate was determined by HPLC and used to calculate cumulative drug release, which was plotted.

Stability of nanoparticles was examined as follows. The prepared pitavastatin nanoparticles were freeze-dried and stored at 4 °C. The average particle size, Zeta potential, and polydispersity index of the nanoparticles were measured at different times.

### Cellular uptake and *in vitro* kinetics of PLGA nanoparticles in EPC

All animal procedures were approved by the Experimental Animal Ethics Committee of the Third Military Medical University before performing the study and conformed to the regulations of Guide for the Care and Use of Laboratory Animals (8th edition, National Research Council, USA, 2011). Cells used in this experiment were extracted using a previously described method^4^ and identified as EPC by their morphology, endothelial cell function, and expression of EPC-specific surface antigen^26^. FITC-PLGA-NPs were prepared as described above. EPC cultured for 1 week were incubated with FITC-NPs (1 mg/ml) for 2 or 4 h, washed three times with PBS, and fixed with 4% paraformaldehyde for 10 min. After staining with 4′,6-diamidino-2-phenylindole and washing three times with PBS, the intracellular distribution of green fluorescence was visualized by laser confocal microscopy (Leica, Germany).

Determination of intracellular drug content for the cellular uptake experiment, the cells were seeded at 1 × 10^6^ cells/well on 6-well culture plates. Then pitavastatin-NPs or pitavastatin (30 μM) was added, the medium was replaced with fresh incubation medium to remove the extracellular drug after 24 h. The cells were collected at fixed time (0 h, 12 h, 24 h, 48 h, 72 h), and lysed using 100 µL RIPA cell lysis solutions (Sigma, U.S.A.), centrifugation at 15,000 rpm for 10 min at 4 °C. Subsequently, 100 µL supernatant was transferred to the new centrifuge tube, 5 µL cell lysate was used to detect the protein content with the bicinchoninic acid (BCA) method. The remainder of the cell lysate was deproteinized using 500 µL methanol under vortex mixing for 1 min and centrifugation again. Supernatant was injected into HPLC vials for anakysis with a fixed injection volume of 50 µL.

### Measurement of NP cytotoxicity

An equal amount of the cell cultured for 1 week suspension (100 μl) was inoculated in each well of a 96-well plate and cultured for 24 h, with each well containing 5 × 10^3^ cells. Different mass concentrations of blank PLGA-NPs (5, 25, 50, 100, and 200 μg/ml) were added to the wells, while cells that were not incubated with blank PLGA NPs served as the control group. Three replicate wells were prepared for each group. The plate was placed in an incubator for 24 h, and 10 μl CCK8 (Beyotime, China) solution were added to each well. After 4 h, absorbance at 450 nm was measured with a microplate reader (EMax, Molecular Devices, U.S.A.).

### Analysis of the effect of pitavastatin-NPs on EPC proliferation *in vitro*

EPC cultured for 1 week were incubated with different concentrations of pitavastatin-NPs and pitavastatin (0.001, 0.01, and 0.1 μM) for 24 h, and then incubated for an additional 72 h with fresh medium followed by digestion with 0.25% trypsin. Cells were centrifuged, counted, and resuspended in Dulbecco’s Modified Eagle’s Medium (DMEM, Gibco, U.S.A.) containing 20% fetal bovine serum (FBS, Gibco, U.S.A.). An equal amount of the cell suspension (100 μl) was inoculated in each well of a 96-well plate and cultured for 24 h; each well contained 5 × 10^3^ cells, with untreated cells serving as the negative control. Three replicate wells were prepared for each group. The plate was placed in an incubator for 24 h, and 10 μl CCK8 solution was added to each well. After 4 h, the absorbance at 450 nm was measured with a microplate reader (EMax, Molecular Devices, U.S.A.).

### Analysis of phosphoinositide 3-kinase (PI3K)/Akt signaling in pitavastatin-NP-mediated biological functions of EPC

EPC cultured for 1 week were divided into blank NP, 0.1 μM pitavastatin-NP, and PI3K inhibitor groups. The last group was pretreated with LY294002 (10 μM, Sigma, U.S.A.) for 30 min before adding pitavastatin-NPs at concentration of 0.1 µM. After 24 h, cells were washed three times with PBS and cultured for 72 h in fresh medium. The subsequent steps were the same as those for determining the effect of pitavastatin-NPs on EPC proliferation.

EPC cultured for 1 week were divided into control (without treatment), 0.1 μM pitavastatin, 0.1 μM pitavastatin-NP, and PI3K signaling pathway inhibitor groups. The last group was pretreated with LY294002 (10 μM) for 30 min followed by 0.1 µM pitavastatin-NPs. After 24 h, cells were collected, total protein was extracted, and the concentration was determined with the bicinchoninic acid assay. After denaturation, proteins (30 μg per well) were separated by 10% sodium dodecyl sulfate polyacrylamide gel electrophoresis and transferred to a polyvinylidene fluoride membrane that was incubated overnight at 4 °C with anti-Akt and anti-phospho- (p-)Akt antibodies (1:500, Cell Signaling technology, U.S.A.). After three 10-min washes with PBS Tween, the membrane was incubated for 1 h at 37 °C with secondary antibody (1:1000), followed by three 10-min washes with PBS Tween. Immunoreactivity was detected by electrochemiluminescence, and images were acquired with a gel imaging system (LAS 4000 mini, GE, U.S.A.). The optical density of each band was measured with Image Quant TL imaging software, and p-Akt/Akt ratio was used as a measure of Akt phosphorylation.

### Evaluation of blood vessel repair by pitavastatin-NPs

A rat carotid artery balloon injury model was established using a non-microsurgical technique^4^. Rats were randomly divided into different groups: surgical injury only, EPC group, 0.01 µM pitavastatin-EPC group, 0.01 µM pitavastatin-NP-EPC group, 0.1 µM pitavastatin-EPC group, 0.1 µM pitavastatin-NP-EPC group. EPC cultured for 7 days were subjected to different treatments for 24 h based on experimental grouping. They were then digested with 0.25% trypsin, collected, and incubated with DiI-acetylated low-density lipoprotein (2.4 μg/ml) for 1 h in the dark, followed by three washes in PBS with centrifugation. EPC (1 × 10^6^ cells) were collected and resuspended in 200 μl saline and immediately injected into rats with carotid artery injury via the tail vein.

EPC homing was evaluated on day 3 post-surgery. Rats were sacrificed by intraperitoneal injection of 2% sodium pentobarbital, and a 5-mm piece of the injured common carotid artery was obtained near the bifurcation and sectioned at a thickness of 7 μm on a cryostat. Sections were labeled with FITC-*Ulex europaeus* agglutinin I (UEA-I). EPC homing and the presence of the endothelial cell phenotype were examined by inverted fluorescence microscope (Leica, Germany).

The re-endothelialization of injured vessels was evaluated on day 7 post-surgery. Rats were anesthetized by intraperitoneal injection of 2% sodium pentobarbital and injected with 5% Evans Blue (25 mg/kg, Sigma, U.S.A.) via the tail vein. The rats were sacrificed 10 min later and a piece of the common carotid artery was obtained near the bifurcation. Blood vessels were sectioned longitudinally and washed with PBS. Areas with complete re-endothelialization were unstained while denuded endothelium with incomplete re-endothelialization was stained blue. Images were acquired and the total vascular and stained areas were measured with Image-Pro Plus 5.1 software (Media Cybernetics Inc., U.S.A.). Percentage vascular re-endothelialization (%) was calculated as (total vascular area − stained area)/total vascular area.

Intimal hyperplasia of injured vessels was evaluated on day 14 post-surgery. Rats were sacrificed by intraperitoneal injection of 2% sodium pentobarbital, and a 5-mm piece of the common carotid artery on the damaged side near the bifurcation was obtained and fixed. After frozen sectioning, the tissue was stained with H&E (Solarbio, China) and visualized and imaged under an inverted microscope (Leica, Germany). Image-Pro Plus 5.1 software (Media Cybernetics Inc., U.S.A.) was used to measure the intimal and medial areas and determine the ratio of intimal to medial areas (I/M).

### Statistical analysis

Data were analyzed with SPSS v.16.0 software (SPSS Inc., Chicago, IL, USA) and are expressed as mean ± standard deviation ( x̅ ± s). Comparisons among multiple groups were performed by one-way analysis of variance, and P < 0.05 indicated that differences were statistically significant.

### Data Availability

The datasets analysed during the current study are available from the corresponding author on reasonable request.
